# Identification of Novel Signal Transduction, Immune Function, and Oxidative Stress Genes and Pathways by Topiramate for Treatment of Methamphetamine Dependence Based on Secondary Outcomes

**DOI:** 10.3389/fpsyt.2017.00271

**Published:** 2017-12-13

**Authors:** Tianhua Niu, Jingjing Li, Ju Wang, Jennie Z. Ma, Ming D. Li

**Affiliations:** ^1^State Key Laboratory for Diagnosis and Treatment of Infectious Diseases, The First Affiliated Hospital, Collaborative Innovation Center for Diagnosis and Treatment of Infectious Diseases, Zhejiang University School of Medicine, Hangzhou, China; ^2^Department of Biochemistry and Molecular Biology, Tulane University School of Medicine, New Orleans, LA, United States; ^3^School of Biomedical Engineering, Tianjin Medical University, Tianjin, China; ^4^Department of Public Health Sciences, University of Virginia, Charlottesville, VA, United States; ^5^Research Center for Air Pollution and Health, Zhejiang University, Hangzhou, China; ^6^Institute of Neuroimmune Pharmacology, Seton Hall University, South Orange, NJ, United States

**Keywords:** topiramate, methamphetamine dependence, gene expression profiling, clinical trial, microarray analysis

## Abstract

**Background:**

Topiramate (TPM) is suggested to be a promising medication for treatment of methamphetamine (METH) dependence, but the molecular basis remains to be elucidated.

**Methods:**

Among 140 METH-dependent participants randomly assigned to receive either TPM (*N* = 69) or placebo (*N* = 71) in a previously conducted randomized controlled trial, 50 TPM- and 49 placebo-treated participants had a total 212 RNA samples available at baseline, week 8, and week 12 time points. Following our primary analysis of gene expression data, we reanalyzed the microarray expression data based on a latent class analysis of binary secondary outcomes during weeks 1–12 that provided a classification of 21 responders and 31 non-responders with consistent responses at both time points.

**Results:**

Based on secondary outcomes, 1,381, 576, 905, and 711 differentially expressed genes at nominal *P* values < 0.05 were identified in responders versus non-responders for week 8 TPM, week 8 placebo, week 12 TPM, and week 12 placebo groups, respectively. Among 1,381 genes identified in week 8 TPM responders, 359 genes were identified in both week 8 and week 12 TPM groups, of which 300 genes were exclusively detected in TPM responders. Of them, 32 genes had nominal *P* values < 5 × 10^−3^ at either week 8 or week 12 and false discovery rates < 0.15 at both time points with consistent directions of gene expression changes, which include *GABARAPL1, GPR155*, and *IL15RA* in GABA receptor signaling that represent direct targets for TPM. Analyses of these 300 genes revealed 7 enriched pathways belonging to neuronal function/synaptic plasticity, signal transduction, inflammation/immune function, and oxidative stress response categories. No pathways were enriched for 72 genes exclusively detected in both week 8 and week 12 placebo groups.

**Conclusion:**

This secondary analysis study of gene expression data from a TPM clinical trial not only yielded consistent results with those of primary analysis but also identified additional new genes and pathways on TPM response to METH addiction.

## Introduction

Methamphetamine (METH), a synthetic derivative of amphetamine, is a highly addictive psychostimulant, with escalating rates of use worldwide (1), particularly in East and Southeast Asia, Australia, various regions of Great Britain, as well as Western and Midwestern areas of United States (1). Due to an additional methyl group in its chemical structure, METH has a higher lipid solubility than the unsubstituted amphetamine, allowing a more rapid transport of the drug across the blood–brain barrier than its parent drug (2). Thus, compared with amphetamine, METH exerts more profound effects on central nervous system (CNS) (3). Approximately 45% of METH is metabolized into amphetamine, and both highly addictive drugs are mostly excreted in renal system (4). The half-life of METH is approximately 8–12 h, and during this time, acute effects of METH take place, which include an ecstatic rush that is nearly immediate in those who either smoke or inject this drug (5). Chronic effects of METH include significant functional impairments in a range of cognitive processes, particularly in attention/psychomotor speed, verbal learning and memory, and fluency-based measures of executive systems functioning (6). Cardiovascular effects of METH are manifested by an elevated heart rate and hypertension, which can result in palpitations, arrhythmias, cardiomyopathy, valvular disease, angina, myocardial infarctions, and cerebral vascular events (7), while psychological effects are manifested by increased anxiety, insomnia, aggression and violent tendencies, paranoia, and visual and auditory hallucinations (8).

Topiramate (TPM), a sulfamate-substituted fructose-1,6-diphosphate analog (9), is a structurally novel anticonvulsant with antiepileptic effects (10). TPM is shown to be efficacious in treating alcohol dependence (11), reducing cocaine intake (12), and facilitating smoking cessation in alcohol-dependent smokers (13). TPM has a long half-life (19–25 h), and has a pharmacokinetic profile linear with dose (in a dose range of 100–800 mg) (14–16). TPM induces an enhancement of GABA_A_-facilitated neuronal activity and concurrently antagonizes ionotropic AMPA and GluR5 kainate glutamate receptor subtypes (17–23), both of which may decrease METH-induced dopamine release in nucleus accumbens. TPM also modulates ionotropic channels by inhibiting L-type calcium channels, limiting the activity of voltage-dependent sodium channels, as well as facilitating potassium conductance (24). Taken together, TPM represents a promising medication for treatment of METH dependence.

To study the efficacy of TPM in treating METH addiction, a multi-center, placebo-controlled randomized trial of 140 METH-dependent subjects was performed (25). This clinical trial generated mixed results such that TPM treatment did not achieve significant METH abstinence (primary outcome), but did decrease weekly median urine METH levels significantly. Genome-wide transcriptomic profiling of peripheral blood (most accessible tissue) has been shown to identify new diagnostic markers for psychiatric disorders (e.g., major depression) (26–28), because of similarities between receptor expression and mechanisms of transduction processes of cells in CNS and in peripheral blood (29). In a previous gene expression study based on the primary outcome of the clinical trial (i.e., weekly abstinence from METH during weeks 1–12) (30), we identified a set of differentially expressed (DE) genes associated with the treatment of TPM on METH dependence, as well as enriched biochemical pathways. However, as shown in the clinical trial, TPM did not significantly increase abstinence from METH use (25), so the numbers of responders with valid gene expression data in the TPM and placebo groups were relatively small, and also were not well balanced (e.g., only four and two responders were included in the placebo group for weeks 8 and 12, respectively) (30). On the other hand, an earlier latent variable analysis study reported by our group (31) showed that the heterogeneity of treatment responses could be better characterized based on six non-longitudinal binary secondary outcomes of METH use, which helped to identify more robust responder and non-responder groups to TPM or placebo, providing larger and more balanced samples. The main objective of this study was to continue a primary analysis of gene expression data based on only primary outcome (30) by conducting a secondary analysis study using the same gene expression data of the same TPM randomized clinical trial (25) but with more samples classified as either responders or non-responders by applying a latent class analysis (LCA) of binary secondary outcomes during weeks 1–12 (31) to obtain a better understanding of the molecular mechanisms associated with the differences between responders and non-responders specifically for TPM.

## Materials and Methods

### Study Participants and Blood Sample Collection

A detailed description of this double-blind, multicenter, placebo-controlled, randomized, parallel-group trial has been presented elsewhere (25). In brief, after a potential participant provided written informed consent, he or she was screened for up to 14 days based on the inclusion and exclusion criteria reported previously (25). If any participant met the eligibility criteria, he or she would start a 14-day baseline assessment. Exclusion criteria included serious medical illness, psychiatric conditions requiring ongoing medication, pregnancy or lactation, nephrolithiasis or renal impairment, and court-mandated drug abuse treatment. For a detailed delineation of screening and baseline assessments, please see our previous report (25). A total 140 Diagnostic and Statistical Manual of Mental Disorders Fourth Edition (DSM-IV)-diagnosed METH-dependent outpatients who were ≥18 years of age who met the eligibility criteria and were qualified for the study were randomized into either TPM (*N* = 69) or placebo (*N* = 71) treatment groups (32). Whole blood was collected in PAXgene Blood RNA tubes (Qiagen, Valencia, CA, USA) from each individual who gave informed content at baseline, week 8, and week 12 time points, respectively. The Institutional Review Boards of each clinical site and the VA Human Rights Committee approved the protocol for conducting of the study and all subjects gave written informed consent in accordance with the Declaration of Helsinki.

### RNA Isolation and Gene Expression Microarray Analysis

A full description has been provided in our primary analysis study reported previously (30). In brief, total RNA was isolated using PAXgene Blood RNA Isolation Kit (Qiagen, Valencia, CA, USA) and stored at −80°C. Transcriptomic profiling was performed by hybridizing each RNA sample to Affymetrix Human Genome U-133 Plus 2.0 arrays (Affymetrix Inc., Santa Clara, CA, USA) by Expression Analysis Inc. (Durham, NC, USA) according to the manufacturer’s protocol. Each array contains 54,675 25-mer probe sets that include approximately 47,000 transcripts and variants out of which 38,500 are well-characterized human genes (33). Gene expression data were then submitted to Information Management Consultants (IMC), Inc. (Reston, VA, USA) for data warehousing using IMC’s Pharmacogenomics Knowledge Management System.

### Classification of Responders and Non-Responders Based on Secondary Outcomes

Subjects were classified by LCA as responders or non-responders according to six *non-longitudinal*, binary secondary outcomes of METH use for weeks 1–12 (31): (i) secondary outcome C for weeks 1–12: ≥21 consecutive days of METH abstinence during weeks 1–12 based on urine drug screens only; (ii) secondary outcome D for weeks 1–12: ≥21 consecutive days of METH abstinence during weeks 1–12 based on urine drug screens and self-report; (iii) secondary outcome E for weeks 1–12: ≥25% reduction in proportion of METH use days during weeks 1–12 compared with self-reported METH use during 14-day baseline period; (iv) secondary outcome F for weeks 1–12: ≥50% reduction in proportion of METH use days during weeks 1–12 compared with self-reported METH use during 14-day baseline period; (v) secondary outcome G for weeks 1–12: ≥25% reduction in median quantitative METH urine concentration during weeks 1–12 compared with median quantitative METH urine concentration during 14-day baseline period; and (vi) secondary outcome H for weeks 1–12: ≥50% reduction in median quantitative METH urine concentration during weeks 1–12 compared with median quantitative METH urine concentration during 14-day baseline period. A detailed description of these six binary secondary outcomes is shown in Supplementary Text S1 in Supplementary Material. These six binary secondary outcomes measured different aspects of both attainment of METH abstinence [(i) and (ii)] and attainment of METH use reduction [(iii), (iv), (v), and (vi)], which could be more robust than primary outcome defined only based on weekly METH abstinence during weeks 1–12 (30) to more sensitively define a responder as a study participant who had decreased METH use during the clinical trial in response to either TPM or placebo. LCA was performed using Mplus (34) to identify clinically distinct classes based on observed response patterns according to the above six secondary outcomes (31).

### Statistical and Bioinformatics Analysis

The procedures used for outlier array detection, quality control, preprocessing, normalization, and probe set filtering to decrease noise have been described before (30). To adjust for individual variations of gene expression at baseline, each individual’s gene expression level at either week 8 or week 12 time point was first baseline-normalized before identification of DE genes and pathways. Then, significantly modulated genes and enriched biological pathways were detected by the following methods.

#### Individual Gene Analysis

Similar to the approach taken by Uusküla et al. (35), two different statistical tests for gene expression analyses were applied: (i) an ordinary Student’s *t*-test, implemented by MATLAB (MathWorks, Natick, MA, USA) and (ii) an empirical Bayes moderated *t*-test, implemented by LIMMA package of Bioconductor, an R-based open-source software (36).

#### Pathway Analysis

Ingenuity Pathway Analysis (IPA)^1^ and Onto-Tools Pathway-Express^2^ software tools were applied. IPA determines the probability that a given gene set is associated with predefined pathways beyond what would be expected by random chance (37). Further, this software tool computes a right-tailed Fisher’s exact test *P* value and a Benjamini–Hochberg false discovery rate (FDR) (38) for each relevant pathway in the Ingenuity Pathways Knowledge Base (IPKB). The list of gene identifiers and their fold changes (FCs) was uploaded to the IPA, such that each gene identifier was mapped to its corresponding gene object in the IPKB. Molecular interaction networks were constructed for both direct and indirect interactions using default parameters. Onto-Tools Pathway-Express (39, 40) is a web-based application that automatically finds significantly impacted Kyoto Encyclopedia of Genes and Genomes (KEGG) pathways. For a given pathway in KEGG that includes *L* input genes, denoted as *g*_1_, …, *g_L_*, Onto-Tools Pathway-Express first calculates a perturbation factor (PF) for gene *i*, denoted as PF(*g_i_*), for *I* ∈ (1, …, *L*), where PF(*g_i_*) shows relative importance of gene *g_i_* for that pathway. Then, an impact factor of that pathway is calculated, which includes PF(*g*_1_), PF(*g*_2_), …, PF(*g_L_*), and a probabilistic term that takes into account the proportion of input genes of that pathway (39). For a given input gene list, Onto-Tools Pathway-Express calculates a gamma *P* value and a Benjamini–Hochberg FDR for each relevant KEGG pathway.

### Data Access

The chip expression data from this study have been deposited in the NCBI database and are accessible through GEO series accession number GSE107015.^3^

## Results

### Description of Responders and Non-Responders Based on Secondary Outcomes

Among the total 140 METH users, 99 (49 in placebo and 50 in TPM group) provided a total 212 chips at baseline (91 chips), week 8 (65 chips), or week 12 (56 chips), of which 209 passed quality control (30). The demographic characteristics of these 99 study participants are presented in Table 1. LCA based on secondary outcomes during weeks 1–12 identified 18 responders and 16 non-responders in week 8 TPM group, and 8 responders and 23 non-responders in week 8 placebo group, 16 responders and 13 non-responders in week 12 TPM group, and 7 responders and 20 non-responders in week 12 placebo group, respectively. However, study subjects could have conflicting efficacy results by being classified as a responder at week 8 but a non-responder at week 12, or *vice versa*. To remove such discrepancies, a responder (or non-responder) was defined as being a responder (or non-responder) consistently at both time points as classified by LCA. Such a restriction made the responder and non-responder groups more homogeneous. Consequently, in this secondary analysis, 15 responders and 12 non-responders with consistent TPM responses at both week 8 and week 12, and 6 responders and 19 non-responders with consistent placebo responses at both time points were included in statistical analysis, which are more balanced than those of primary analysis: week 8 TPM group (5 responders and 17 non-responders), week 8 placebo group (4 responders and 17 non-responders), week 12 TPM group (6 responders and 11 non-responders), and week 12 placebo group (2 responders and 13 non-responders), and had consistently greater total sample sizes for these 4 respective groups.

**Table 1:** Demographic characteristics of 99 study participants with available gene expression microarrays.

Trial group		Gender		Race		Ethnicity	
Placebo	49	F	15	Asian, Black, or mixed	2	Hispanic	0
						Non-Hispanic	2
				White	13	Hispanic	1
						Non-Hispanic	12^a^

		M	34	Asian, Black, or mixed	7	Hispanic	0
						Non-Hispanic	7
				White	27	Hispanic	4
						Non-Hispanic	23

Topiramate	50	F	19	Asian, Black, or mixed	2	Hispanic	0
						Non-Hispanic	2
				White	17	Hispanic	1
						Non-Hispanic	16

		M	31	Asian, Black, or mixed	2	Hispanic	0
						Non-Hispanic	2
				White	29	Hispanic	4
						Non-Hispanic	25^a^

*^a^Including an “Unknown” Ethnicity in the “White” Group*.

### Identification of DE Genes in Treatment Responders

We applied two different tests, i.e., an ordinary Student’s *t*-test and an empirical Bayes moderated *t*-test, for detecting DE genes (Table S1 in Supplementary Material). By applying an ordinary Student’s *t*-test, based on a nominal *P* value < 0.05, we identified 1,186, 513, 758, and 611 genes for week 8 TPM, week 8 placebo, week 12 TPM, and week 12 placebo, respectively. By applying an empirical Bayes moderated *t*-test, based on a nominal *P* value < 0.05, we identified 759, 145, 388, and 286 genes for these respective groups, respectively (Tables S2–S5 in Supplementary Material). A total of 564 (74.31%), 82 (56.55%), 241 (62.11%), and 186 (65.03%) genes (using the number of genes detected by the empirical Bayes moderated *t*-test as a denominator) for week 8 TPM, week 8 placebo, week 12 TPM, and week 12 placebo were also detected by the ordinary Student’s *t*-test. Because the proportion of overlap is greater than 50% for each of these four groups, genes detected by both tests were pooled together, such that if a gene is selected by either ordinary Student’s *t*-test or empirical Bayes moderated *t*-test, and if a gene is detected by both tests, then the test with the smaller nominal *P* value is chosen along with the corresponding FC and FDR. Together, there are 1,381 (FDR: 0.16 ± 0.059) (Table S2 in Supplementary Material), 576 (FDR: 0.36 ± 0.094) (Table S3 in Supplementary Material), 905 (FDR: 0.25 ± 0.090) (Table S4 in Supplementary Material), and 711 (FDR: 0.29 ± 0.052) (Table S5 in Supplementary Material) for the above four groups, respectively. To identify more likely biologically meaningful DE genes, similar to primary analysis (30), we applied a more stringent statistical significance threshold, i.e., a nominal *P* value < 0.01, which revealed 380 (FDR: 0.12 ± 0.064), 123 (FDR: 0.30 ± 0.075), 199 (FDR: 0.18 ± 0.095), and 122 (FDR: 0.26 ± 0.060) genes for week 8 TPM, week 8 placebo, week 12 TPM, and week 12 placebo groups, respectively. Of them, week 8 TPM group had the lowest average FDR (0.12), followed by week 12 TPM (0.18), week 8 placebo (0.30), and week 12 placebo (0.26), and these results are better than the results obtained from primary analysis (30), which had an increasing order of week 8 TPM (0.009), week 12 placebo (0.033), week 8 placebo (0.027), and week 12 TPM (0.113), because week 12 TPM group of primary outcome had fewer (total: 17) and more imbalanced (6 responders versus 11 non-responders) samples than week 12 TPM group of secondary outcomes (total: 27, 15 responders versus 12 non-responders). Further, 298, 0, 112, and 10 of the above genes with nominal *P* values < 0.01 also had FDRs < 0.15 for the above four groups, showing that many more genes’ expressions were significantly changed by TPM than by placebo at each time point, with control for multiple testing. Of 359 genes shared between week 8 TPM and week 12 TPM groups, 300 genes were exclusively detected in TPM responders. Of 106 genes shared between week 8 placebo and week 12 placebo groups, 72 genes were exclusively detected in placebo responders. There is no overlap between these two gene sets.

Among 300 genes exclusively detected in week 8 and week 12 TPM responders, 34 genes had nominal *P* values < 5 × 10^−3^ at either time point and FDRs < 0.15 at both time points. Of them, two genes, *FNIP2* (week 8 TPM Group: FC ± SD = 1.49 ± 0.20, *P* value = 0.022, FDR = 0.13; week 12 TPM Group: FC ± SD = −1.28 ± 0.10, *P* value = 0.0032, FDR = 0.13), and *TOX4* (week 8 TPM Group: FC ± SD = −1.23 ± 0.090, *P* value = 0.010, FDR = 0.12; week 12 TPM Group: FC ± SD = 1.28 ± 0.098, *P* value = 0.0038, FDR = 0.14) had inconsistent directions of gene expression changes, and were subsequently excluded. Therefore, 32 genes (17 down- and 15 upregulated) were selected based on the above statistics criteria showing consistent directions of gene expression changes at both time points (Table 2), which included 12 biologically important genes for drug addiction: *CASP4, COX19, CUX1, GABARAPL1, GNG2, GPR155, HSF1, IL15RA, NLRP1, SIL1, SLC25A19*, and *UBAP2*. Also, as shown in Table 2, 12 genes have statistical support from both tests for both week 8 TPM and week 12 TPM groups: *ASXL1, CASP4, COX19, FBXL13, GABARAPL1, GPR155, IL15RA, LUZP1, PTCD1, SLC25A19, SUV39H1*, and *UBAP2*. Of them, *GABARAPL1, GPR155*, and *IL15RA* in GABA receptor signaling represent direct targets for TPM. By contrast, none of 72 genes exclusively detected in week 8 and week 12 placebo responders contain direct targets for TPM.

**Table 2:** A list of 32 representative genes significantly and consistently modulated exclusively in weeks 8 and 12 topiramate (TPM) groups (*n* = 32) based on secondary outcomes.^a^

Gene symbol	Gene name	Week 8 TPM			Week 12 TPM		
		FC ± SD^b^	*P* value	FDR^c^	FC ± SD^b^	*P* value	FDR^c^
**Synaptic plasticity ands nervous system development/function**							
*GABARAPL1*	GABA(A) receptor-associated protein like 1	**1.44 ± 0.17**	**3.43 × 10^−3^**	**0.096**	**1.48 ± 0.17**	**2.04 × 10^−3^**	**0.13**
*NLRP1*	NLR family, pyrin domain containing 1	1.17 ± 0.07	0.031	0.14	1.24 ± 0.07	4.04 × 10^−3^	0.11
*TMEM55B*	Transmembrane protein 55B	−1.14 ± 0.06	0.035	0.15	−1.15 ± 0.04	2.95 × 10^−3^	0.13

**Signal transduction**							
*CASP4*	Caspase 4, apoptosis-related cysteine peptidase	**1.25 ± 0.11**	**0.0178**	**0.13**	1.39 ± 0.14	**2.75 × 10^−3^**	**0.13**
*CSNK1A1*	Casein kinase 1, alpha 1	1.17 ± 0.07	0.016	0.12	1.32 ± 0.11	4.07 × 10^−3^	0.14
*GIMAP7*	GTPase, IMAP family member 7	**1.50 ± 0.24**	**0.0150**	**0.12**	1.43 ± 0.14	1.15 × 10^−3^	0.12
*GNG2*	Guanine nucleotide binding protein (G protein), gamma 2	**1.43 ± 0.20**	**0.0217**	**0.13**	1.46 ± 0.15	1.41 × 10^−3^	0.12
*GPR155*	G-protein-coupled receptor 155	**1.25 ± 0.10**	**7.15 × 10^−3^**	**0.11**	**1.69 ± 0.19**	**1.47 × 10^−4^**	**0.075**
*HN1*	Hematological and neurological expressed 1	1.14 ± 0.06	0.037	0.15	**1.15 ± 0.04**	**8.40 × 10^−4^**	**0.12**
*INPP5B*	Inositol polyphosphate-5-phosphatase, 75 kDa	**−1.34 ± 0.09**	**2.24 × 10^−3^**	**0.086**	−1.24 ± 0.07	4.23 × 10^−4^	0.11

**Ubiquitination/intracellular protein transport**							
*FBXL13*	F-box and leucine-rich repeat protein 13	**1.36 ± 0.11**	**2.72 × 10^−3^**	**0.090**	**1.70 ± 0.26**	**3.80 × 10^−3^**	**0.14**
*SIL1*	SIL1 homolog, endoplasmic reticulum chaperone (*S. cerevisiae*)	−1.23 ± 0.10	9.92 × 10^−3^	0.12	**−1.35 ± 0.10**	**5.93 × 10^−4^**	**0.12**
*UBAP2*	Ubiquitin associated protein 2	**−1.23 ± 0.10**	**0.023**	**0.13**	**−1.24 ± 0.09**	**4.30 × 10^−3^**	**0.15**

**Mitochondrial function/metabolism and energy pathways**							
*ASRGL1*	Asparaginase like 1	−1.24 ± 0.09	0.019	0.13	**−1.37 ± 0.12**	**1.62 × 10^−3^**	**0.13**
*COX19*	COX19 cytochrome *c* oxidase assembly homolog (*S. cerevisiae*)	**1.25 ± 0.10**	**8.33 × 10^−3^**	**0.11**	**1.28 ± 0.10**	**2.29 × 10^−3^**	**0.13**
*DECR1*	2,4-Dienoyl CoA reductase 1, mitochondrial	1.17 ± 0.07	0.022	0.13	1.21 ± 0.06	1.70 × 10^−3^	0.13
*PPME1*	Protein phosphatase methylesterase 1	−1.16 ± 0.05	1.41 × 10^−3^	0.081	−1.17 ± 0.05	2.38 × 10^−3^	0.13
*PTCD1*	Pentatricopeptide repeat domain 1	**−1.20 ± 0.06**	**1.71 × 10^−3^**	**0.082**	**−1.26 ± 0.06**	**1.81 × 10^−4^**	**0.075**
*SLC25A19*	Solute carrier family 25 (mitochondrial thiamine pyrophosphate carrier), member 19	**−1.15 ± 0.05**	**4.52 × 10^−3^**	**0.10**	**−1.29 ± 0.09**	**6.97 × 10^−4^**	**0.12**

**Transcriptional regulation**							
*ASXL1*	Additional sex combs like 1 (*Drosophila*)	**−1.25 ± 0.08**	**2.06 × 10^−3^**	**0.085**	**−1.27 ± 0.05**	**1.30 × 10^−5^**	**0.047**
*CPSF3L*	Cleavage and polyadenylation specific factor 3-like	**−1.21 ± 0.09**	**0.013867**	**0.12**	−1.21 ± 0.07	3.90 × 10^−3^	0.14
*CUX1*	Cut-like homeobox 1	−1.32 ± 0.13	0.011	0.12	−1.34 ± 0.10	3.33 × 10^−3^	0.13
*HSF1*	Heat shock transcription factor 1	**−1.19 ± 0.07**	**7.78 × 10^−3^**	**0.11**	−1.26 ± 0.09	2.92 × 10^−3^	0.13
*JARID1A*	Jumonji, AT rich interactive domain 1A	1.40 ± 0.22	0.031	0.141	**1.55 ± 0.21**	**0.002653**	**0.13**
*LUZP1*	Leucine zipper protein 1	**−1.22 ± 0.09**	**6.75 × 10^−3^**	**0.11**	**−1.29 ± 0.09**	**2.54 × 10^−3^**	**0.13**
*SNRPB*	Small nuclear ribonucleoprotein polypeptides B and B1	**−1.20 ± 0.07**	**7.30 × 10^−3^**	**0.11**	−1.23 ± 0.08	2.93 × 10^−3^	0.13
*SUV39H1*	Suppressor of variegation 3–9 homolog 1 (*Drosophila*)	**−1.34 ± 0.10**	**7.07 × 10^−4^**	**0.066**	**−1.32 ± 0.10**	**8.87 × 10^−4^**	**0.12**
*ZNF354A*	Zinc finger protein 354A	**1.32 ± 0.12**	**3.63 × 10^−3^**	**0.097**	1.46 ± 0.16	2.83 × 10^−3^	0.13

**Immune system function**							
*IL15RA*	Interleukin 15 receptor, alpha	**−1.23 ± 0.09**	**5.96 × 10^−3^**	**0.11**	**−1.22 ± 0.06**	**9.74 × 10^−4^**	**0.12**

**Other**							
*ARPC3*	Actin related protein 2/3 complex, subunit 3, 21 kDa	1.20 ± 0.08	8.10 × 10^−3^	0.11	1.21 ± 0.07	2.79 × 10^−3^	0.13
*COMMD4*	COMM domain containing 4	**−1.18 ± 0.06**	**4.07 × 10^−3^**	**0.097**	−1.21 ± 0.07	1.27 × 10^−3^	0.12
*ZC3H7B*	Zinc finger CCCH-type containing 7B	1.35 ± 0.16	0.023	0.13	**1.40 ± 0.15**	**4.72 × 10^−3^**	**0.15**

*^a^Genes were selected from a total of 300 genes detected exclusively for both week 8 and week 12 TPM groups (at a nominal *P* value threshold of 0.05), with a nominal *P* value < 5 × 10^−3^ for either group and false discovery rates (FDRs) < 0.15 for both groups. Genes in each function category were sorted by an alphabetical order. If a gene was detected by both ordinary Student’s *t*-test and empirical Bayes moderated *t*-test at a nominal *P* value < 0.05, that gene’s numerical values were highlighted in bold font*.

*^b^Fold change (FC) is defined as the ratio of the baseline-corrected expression values of responders over non-responders*.

*^c^FDR was estimated by the Benjamini–Hochberg method*.

By comparing these above DE genes detected at nominal *P* values < 0.01 based on secondary outcomes with those DE detected at same statistical significance threshold based on primary outcome for week 8 TPM, week 8 placebo, week 12 TPM, and week 12 placebo groups, respectively (30), 55, 11, 11, and 15 genes were shared between primary analysis and secondary analysis for the above four groups, respectively (Table 3). Of them, *SASH1* was detected as a downregulated gene in both week 8 TPM and week 12 TPM groups, and there were no other overlapping genes shared between the two time points for TPM response. The directions of gene expression changes between primary analysis and secondary analysis were different for 8 genes (8.89%) of these 90 unique genes, i.e., *TNRC6A* and *USP16* for week 8 TPM, *RBMS1* and *WDR68* for week 12 TPM, *EIF4B* and *NACA* for week 8 placebo, and *FBXL11* and *ITM2A* for week 12 placebo group, respectively (Table 3). It is noteworthy that 9 of the 55 genes identified by primary analysis and secondary analysis for week 8 TPM—including 4 upregulated genes, i.e., *CD164, AKAP11, FGFR1OP2*, and *PTEN*, and 5 downregulated genes, i.e., *EMILIN2, DGCR14, BCR, GANAB*, and *NAGK*, were among the 93 (48 up- and 45 downregulated) representative genes selected based on primary analysis (30), and the directions of gene expression changes were all consistent between these two analyses.

**Table 3:** A list of significantly modulated genes detected in both primary analysis and secondary analysis (*n* = 92).^a^

Gene symbol	Gene name	Primary analysis			Secondary analysis		
		FC ± SD^b^	*P* value	False discovery rate (FDR)^c^	FC ± SD^b^	*P* value	FDR^c^
**Week 8 topiramate (TPM) (***n*** = 55)**							

*ACO2	Aconitase 2, mitochondrial	−1.20 ± 0.06	2.15 × 10^−3^	0.011	**−1.31 ± 0.10**	**0.0011**	**0.24**
*AGPAT3*	1-Acylglycerol-3-phosphate *O*-acyltransferase 3	−1.19 ± 0.05	8.42 × 10^−4^	0.0057	**−1.21 ± 0.07**	**0.0029**	**0.095**
*AKAP11*	A kinase (PRKA) anchor protein 11	2.23 ± 0.29	1.10 × 10^−5^	2.64 × 10^−4^	1.31 ± 0.12	0.0072	0.11
*ANKRD10*	Ankyrin repeat domain 10	1.32 ± 0.11	4.33 × 10^−3^	0.0188	1.41 ± 0.18	0.0092	0.12
*BCR*	Breakpoint cluster region	−1.43 ± 0.06	<1 × 10^−6^	<1 × 10^−5^	**−1.21 ± 0.07**	**0.0034**	**0.096**
*CD164*	CD164 molecule, sialomucin	2.67 ± 0.38	1.00 × 10^−6^	5.07 × 10^−5^	1.54 ± 0.20	0.0090	0.12
*CDK9	Cyclin-dependent kinase 9	−1.53 ± 0.18	7.99 × 10^−3^	0.030	**−1.54 ± 0.15**	**8.44 × 10^−5^**	**0.24**
*CLK4*	CDC-like kinase 4	1.84 ± 0.21	7.90 × 10^−5^	0.0010	1.69 ± 0.30	0.0095	0.12
*CTSA	Cathepsin A	−1.43 ± 0.14	0.005526	0.023	**−1.34 ± 0.12**	**0.0029**	**0.24**
*DENND1A*	DENN/MADD domain containing 1A	−1.72 ± 0.23	0.006197	0.024668	−1.39 ± 0.11	0.0011	0.078
*DGCR14*	DiGeorge syndrome critical region gene 14	−1.45 ± 0.04	<1 × 10^−6^	<1 × 10^−5^	**−1.26 ± 0.09**	**0.0025**	**0.089**
*DIAPH1*	Diaphanous homolog 1 (*Drosophila*)	−1.28 ± 0.09	0.0029	0.014	**−1.20 ± 0.06**	**0.0038**	**0.097**
*EMILIN2	Elastin microfibril interfacer 2	−1.41 ± 0.05	<1 × 10^−6^	<1 × 10^−5^	**−1.33 ± 0.13**	**0.0073**	**0.24**
*FGFR1OP2*	FGFR1 oncogene partner 2	1.57 ± 0.11	4.00 × 10^−6^	1.31 × 10^−4^	1.35 ± 0.14	0.0082	0.11
*FLNA	Filamin A, alpha (actin binding protein 280)	−2.26 ± 0.49	0.0076	0.029	**−1.64 ± 0.27**	**0.0046**	**0.24**
*GAA*	Glucosidase, alpha; acid	−1.5 ± 0.13	2.36 × 10^−4^	0.0023	−1.39 ± 0.16	0.0095	0.12
*GAK*	Cyclin G associated kinase	−1.24 ± 0.05	2.13 × 10^−4^	0.002176	−1.15 ± 0.05	0.0085	0.11
*GANAB	Glucosidase, alpha; neutral AB	−1.63 ± 0.08	<1 × 10^−6^	<1 × 10^−5^	−1.42 ± 0.16	0.0044	0.24
*GRN*	Granulin	−1.36 ± 0.13	0.0045	0.019	**−1.32 ± 0.13**	**0.0087**	**0.12**
*HSPBAP1*	HSPB (heat shock 27 kDa) associated protein 1	1.49 ± 0.15	0.0032	0.015	1.33 ± 0.11	0.0019	0.085
*JUND*	Jun D proto-oncogene	−1.24 ± 0.05	4.50 × 10^−5^	7.08 × 10^−4^	−1.25 ± 0.08	0.0021	0.086
*LRRC41*	Leucine-rich repeat containing 41	−1.35 ± 0.11	0.0039	0.017	**−1.31 ± 0.09**	**4.31 × 10^−4^**	**0.060**
*MALAT1	Metastasis associated lung adenocarcinoma transcript 1 (non-protein coding)	3.25 ± 0.78	4.52 × 10^−5^	0.0037	2.17 ± 0.58	0.0066	0.24
*MED12	Mediator complex subunit 12	−1.48 ± 0.17	0.0044	0.019	**−1.43 ± 0.16**	**0.0028**	**0.24**
*MED25*	Mediator complex subunit 25	−1.68 ± 0.17	1.55 × 10^−4^	0.0017	−1.67 ± 0.27	0.0087	0.12
*MED26*	Mediator complex subunit 26	−1.16 ± 0.05	0.0030	0.014	**−1.22 ± 0.06**	**6.21 × 10^−4^**	**0.066**
*METTL9	Methyltransferase like 9	1.33 ± 0.06	1.00 × 10^−5^	2.53 × 10^−4^	**1.27 ± 0.10**	**0.0061**	**0.24**
*NAGK*	*N*-acetylglucosamine kinase	−1.3 ± 0.05	4.00 × 10^−6^	1.31 × 10^−4^	−1.15 ± 0.05	0.0065	0.11
*NFE2L1*	Nuclear factor (erythroid-derived 2)-like 1	−1.17 ± 0.04	6.40 × 10^−5^	8.93 × 10^−4^	−1.14 ± 0.05	0.0084	0.11
*PARVB*	Parvin, beta	−1.74 ± 0.22	0.0021	0.011	**−1.51 ± 0.20**	**0.0038**	**0.097**
*PIK3C2A*	Phosphoinositide-3-kinase, class 2, alpha polypeptide	2.05 ± 0.24	2.30 × 10^−5^	4.41 × 10^−4^	**1.2 ± 0.07**	**0.0073**	**0.11**
*PIM1*	Pim-1 oncogene	−1.26 ± 0.07	0.0011	0.0069	−1.24 ± 0.07	0.0021	0.085
*PLCG1*	Phospholipase C, gamma 1	−1.27 ± 0.05	3.30 × 10^−5^	5.68 × 10^−4^	−1.37 ± 0.13	0.0049	0.10
*PML	Promyelocytic leukemia	−1.51 ± 0.18	0.0084	0.031	**−1.44 ± 0.13**	**3.15 × 10^−4^**	**0.24**
*POLDIP2*	Polymerase (DNA directed), delta interacting protein 2	−1.48 ± 0.14	0.0081	0.030	−1.25 ± 0.07	0.0012	0.079
*POLR2E*	Polymerase (RNA) II (DNA directed) polypeptide E, 25 kDa	−1.38 ± 0.12	0.0067	0.026	−1.22 ± 0.07	7.53 × 10^−4^	0.068
*PPME1*	Protein phosphatase methylesterase 1	−1.18 ± 0.05	0.0042	0.018	−1.16 ± 0.05	0.0014	0.081
*PRPF19*	PRP19/PSO4 pre-mRNA processing factor 19 homolog (*S. cerevisiae*)	−1.42 ± 0.11	3.07 × 10^−4^	0.0028	**−1.48 ± 0.18**	**0.0040**	**0.097**
*PTEN*	Phosphatase and tensin homolog	2.01 ± 0.10	<1 × 10^−6^	<1 × 10^−5^	1.30 ± 0.12	0.0053	0.10
*RAB35*	RAB35, member RAS oncogene family	−1.34 ± 0.10	0.0083	0.030	−1.25 ± 0.10	0.0097	0.12
*RNH1	Ribonuclease/angiogenin inhibitor 1	−1.44 ± 0.14	0.0042	0.018	**−1.34 ± 0.12**	**0.0019**	**0.24**
*RSF1*	Remodeling and spacing factor 1	−1.27 ± 0.09	0.0041	0.018	−1.47 ± 0.21	0.0089	0.12
*SASH1	SAM and SH3 domain containing 1	−1.49 ± 0.17	0.0038	0.017	**−1.49 ± 0.17**	**0.0017**	**0.24**
*SBF1*	SET binding factor 1	−1.64 ± 0.08	<1 × 10^−6^	<1 × 10^−5^	**−1.27 ± 0.09**	**0.0027**	**0.090**
*SCAND1*	SCAN domain containing 1	−1.52 ± 0.17	0.0062	0.025	−1.41 ± 0.11	3.41 × 10^−4^	0.058
*SMARCD2*	SWI/SNF related, matrix associated, actin dependent regulator of chromatin, subfamily d, member 2	−1.47 ± 0.09	2.73 × 10^−4^	0.0026	−1.25 ± 0.09	0.0097	0.12
*SUV39H1*	Suppressor of variegation 3–9 homolog 1 (*Drosophila*)	−1.51 ± 0.08	1.00 × 10^−6^	5.07 × 10^−6^	**−1.34 ± 0.10**	**7.07 × 10^−4^**	**0.066**
*TNRC6A*^d^	Trinucleotide repeat containing 6A	1.38 ± 0.12	0.0016	0.0093	−1.34 ± 0.12	0.0071	0.11
*TPP1*	Tripeptidyl peptidase I	−1.49 ± 0.12	7.80 × 10^−5^	0.0010	**−1.48 ± 0.14**	**0.0016**	**0.082**
*TRAPPC9*	Trafficking protein particle complex 9	−1.46 ± 0.14	0.0040	0.018	−1.32 ± 0.11	0.0024	0.087
*UBR5*	Ubiquitin protein ligase E3 component n-recognin 5	1.43 ± 0.1	1.08 × 10^−4^	0.0013	1.49 ± 0.20	0.0072	0.11
*USP16*^d^	Ubiquitin specific peptidase 16	1.67 ± 0.23	0.0028	0.014	−1.97 ± 0.31	0.0032	0.096
*VISA*	Virus-induced signaling adapter	−1.27 ± 0.09	0.0041	0.018	−1.36 ± 0.06	1.00 × 10^−6^	0.0039
*ZNF12*	Zinc finger protein 12	1.9 ± 0.23	1.09 × 10^−4^	0.0013	1.74 ± 0.25	0.0083	0.11
*ZNF207*	Zinc finger protein 207	1.32 ± 0.09	5.54 × 10^−4^	0.0042	1.4 ± 0.17	0.0093	0.12

**Week 8 placebo (***n*** = 11)**							

*DNLZ*	DNL-type zinc finger	−1.40 ± 0.11	9.82 × 10^−4^	0.016	−1.49 ± 0.12	0.0021	0.28
*EIF4B*^d^	Eukaryotic translation initiation factor 4B	1.22 ± 0.07	0.0038	0.039	−1.29 ± 0.16	0.0055	0.30
*MAP4*	Microtubule-associated protein 4	1.25 ± 0.09	0.0079	0.060	1.35 ± 0.11	0.0050	0.30
*NACA*^d^	Nascent polypeptide-associated complex alpha subunit	1.15 ± 0.03	3.2 × 10^−5^	0.0021	−1.12 ± 0.04	0.0081	0.31
*NUP93*	Nucleoporin 93 kDa	1.39 ± 0.08	3.50 × 10^−5^	0.0022	1.5 ± 0.07	7.46 × 10^−4^	0.23
*PEX16*	Peroxisomal biogenesis factor 16	−1.25 ± 0.07	0.0010	0.017	−1.25 ± 0.06	3.25 × 10^−4^	0.17
*PSAP*	Prosaposin	1.25 ± 0.08	0.0054	0.047	1.51 ± 0.34	0.0067	0.30
*PSME3*	Proteasome (prosome, macropain) activator subunit 3 (PA28 gamma; Ki)	1.29 ± 0.08	0.0056	0.049	1.34 ± 0.11	0.0073	0.31
*SMARCA2*	SWI/SNF related, matrix associated, actin dependent regulator of chromatin, subfamily a, member 2	1.81 ± 0.16	2.30 × 10^−5^	0.0017	1.51 ± 0.20	1.4 × 10^−4^	0.16
*SMARCC1*	SWI/SNF related, matrix associated, actin dependent regulator of chromatin, subfamily c, member 1	1.21 ± 0.05	3.39 × 10^−4^	0.0083	1.46 ± 0.20	0.0010	0.27
*XPO5*	Exportin 5	1.37 ± 0.07	1.08 × 10^−4^	0.0042	1.32 ± 0.06	0.0070	0.30

**Week 12 TPM (***n*** = 11)**							

*ATP8B1*	ATPase, class I, type 8B, member 1	1.51 ± 0.16	0.0027	0.10	**1.66 ± 0.27**	**0.0044**	**0.15**
*ERAP1*	Endoplasmic reticulum aminopeptidase 1	−1.43 ± 0.11	0.0026	0.10	−1.27 ± 0.11	0.0098	0.20
*HNRNPA3*	Heterogeneous nuclear ribonucleoprotein A3	1.25 ± 0.09	0.0077	0.14	1.26 ± 0.10	0.0093	0.20
*IL15RA*	Interleukin 15 receptor, alpha	−1.33 ± 0.12	0.0089	0.16	**−1.22 ± 0.06**	**9.74 × 10^−4^**	**0.12**
*POLA2*	Polymerase (DNA directed), alpha 2 (70 kDa subunit)	−1.4 ± 0.14	0.0096	0.16	−1.31 ± 0.12	0.0066	0.39
*RBMS1*^d^	RNA binding motif, single stranded interacting protein 1	−1.23 ± 0.06	9.47 × 10^−4^	0.079	1.32 ± 0.10	0.0040	0.14
*SASH1*	SAM and SH3 domain containing 1	−1.75 ± 0.23	0.0026	0.10	−1.87 ± 0.18	6.90 × 10^−5^	0.073
*SIGLEC10*	Sialic acid binding Ig-like lectin 10	−1.3 ± 0.08	0.0024	0.10	−1.19 ± 0.07	0.0096	0.20
*SIL1*	SIL1 homolog, endoplasmic reticulum chaperone (*S. cerevisiae*)	−1.28 ± 0.1	0.0069	0.14	−1.35 ± 0.10	5.93 × 10^−4^	0.12
*TCEB3*	Transcription elongation factor B (SIII), polypeptide 3 (110 kDa, elongin A)	−1.45 ± 0.12	9.85 × 10^−4^	0.079	−1.32 ± 0.12	0.0028	0.13
*WDR68*^d^	WD repeat domain 68	1.50 ± 0.11	1.35 × 10^−4^	0.045	−1.29 ± 0.10	0.0040	0.14

**Week 12 placebo (***n*** = 15)**							

*BCL2L1*	BCL2-like 1	2.14 ± 0.26	3.30 × 10^−5^	0.0031	2.27 ± 0.41	8.45 × 10^−4^	0.20
*DYNC1H1*	Dynein, cytoplasmic 1, heavy chain 1	1.28 ± 0.07	0.0015	0.027	1.56 ± 0.18	0.0057	0.27
*FBXL11*^d^	F-box and leucine-rich repeat protein 11	1.30 ± 0.09	0.0083	0.075	−1.31 ± 0.06	0.0067	0.27
*HPS1*	Hermansky–Pudlak syndrome 1	1.61 ± 0.13	2.54 × 10^−4^	0.010	1.70 ± 0.12	0.0091	0.27
*HYAL2*	Hyaluronoglucosaminidase 2	−1.48 ± 0.07	0.0063	0.062	−1.3 ± 0.08	9.79 × 10^−4^	0.21
*IDH3A*	Isocitrate dehydrogenase 3 (NAD+) alpha	1.19 ± 0.05	9.99 × 10^−3^	0.084	−1.36 ± 0.14	0.0083	0.27
*IMP3*	IMP3, U3 small nucleolar ribonucleoprotein, homolog (yeast)	−1.19 ± 0.06	0.0044	0.050	−1.42 ± 0.20	0.0018	0.27
*ITM2A*^d^	Integral membrane protein 2A	−1.30 ± 0.1	0.0052	0.056	1.24 ± 0.308	0.0081	0.27
*LILRA5	Leukocyte immunoglobulin-like receptor, subfamily A (with TM domain), member 5	−1.59 ± 0.21	0.0053	0.057	−2.56 ± 0.84	0.0067	0.38
*NDUFS8*	NADH dehydrogenase (ubiquinone) Fe-S protein 8, 23 kDa (NADH-coenzyme Q reductase)	−1.84 ± 0.26	0.0052	0.056	−1.41 ± 0.20	0.0058	0.27
*PCGF5*	Polycomb group ring finger 5	1.4 ± 0.10	6.98 × 10^−4^	0.018	1.55 ± 0.08	1.67 × 10^−4^	0.11
*PDCD4*	Programmed cell death 4 (neoplastic transformation inhibitor)	−1.27 ± 0.07	0.0030	0.040	−1.42 ± 0.16	0.0071	0.27
*RNF123*	Ring finger protein 123	2.25 ± 0.25	9.31 × 10^−4^	0.020	1.82 ± 0.29	0.0089	0.27
*TOB1	Transducer of ERBB2, 1	1.64 ± 0.16	0.0010	0.021	**1.96 ± 0.67**	**0.0027**	**0.38**
*TSPAN5*	Tetraspanin 5	2.20 ± 0.39	0.0032	0.042	1.99 ± 0.50	0.0043	0.27

*^a^For primary analysis, genes were selected only based on the ordinary Student’s *t*-test at nominal *P* values < 0.01 (30). For secondary analysis, genes were selected based on either the ordinary Student’s *t*-test (without an asterisk) or the empirical Bayes moderated *t*-test (with an asterisk) at nominal *P* values < 0.01. Genes within each treatment category were sorted by an alphabetical order. For secondary analysis, a gene was detected by both ordinary Student’s *t*-test and empirical Bayes moderated *t*-test at a nominal *P* value < 0.01, that gene’s numerical values were highlighted in bold font*.

*^b^Fold change (FC) is defined as the ratio of the baseline-corrected expression values of responders over non-responders*.

*^c^FDR was estimated by the Benjamini–Hochberg method*.

*^d^The directions of gene expression changes were different between primary outcome and secondary outcomes groups*.

**Figure 1:**
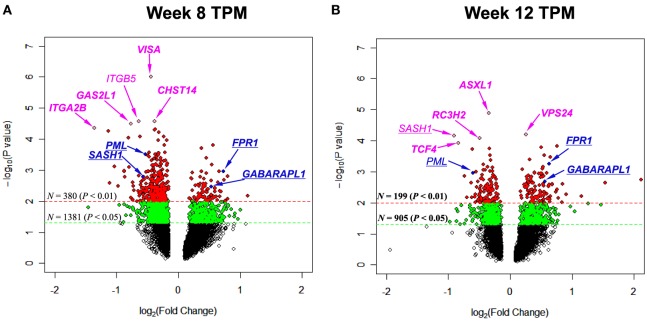
Volcano plots depicting log_2_(Fold Change) (*x*-axis) and −log_10_(*P* value) (*y*-axis) for genes of **(A)** week 8 topiramate (TPM) and **(B)** week 12 TPM groups. Genes with 0.01 ≤ *P* values < 0.05 and *P* values < 0.01 were shown by green and red colors, respectively. Five most statistically significant genes for each group were shown in pink color. Genes with *P* values < 5 × 10^−3^ and |Fold Change|s > 1.40 for both week 8 and week 12 TPM groups were underlined, and shown in blue color [except in **(B)**, *SASH1* was shown in pink color, because this gene was among top five]. For each group, if a gene was detected by both ordinary Student’s *t*-test and empirical Bayes moderated *t*-test at a nominal *P* value < 0.05, that gene’s corresponding symbol was highlighted in bold font. **(A)** *ITGB5* is not in bold font, and all other gene symbols were in bold font; **(B)** *SASH1* and *PML* are not in bold font, and all other gene symbols were in bold font.

To pinpoint top genes that were changed in TPM responders compared with TPM non-responders, volcano plots, where the log_10_(*P* value)’s are plotted versus log_2_(FC)’s, were generated for week 8 TPM (Figure 1A) and week 12 TPM group (Figure 1B), respectively. In week 8 TPM group, the top five genes (pink color) changed in TPM responders compared with TPM non-responders were *VISA* (*P* value = 1.00 × 10^−6^, FC = −1.36, FDR = 0.0039), *CHST14* (*P* value = 2.70 × 10^−5^, FC = −1.30, FDR = 0.026), *ITGB5* (*P* value = 2.70 × 10^−5^, FC = −1.56, FDR = 0.026), *GAS2L1* (*P* value = 3.20 × 10^−5^, FC = −1.70, FDR = 0.026), and *ITGA2B* (*P* value = 4.50 × 10^−5^, FC = −2.56, FDR = 0.026) (i.e., top five genes shown in Table S2 in Supplementary Material). Of them, none was significantly changed in week 8 placebo group at a nominal *P* value < 0.01. In week 12 TPM group, the top five genes (pink color) changed in TPM responders compared with TPM non-responders were *ASXL1* (*P* value = 1.30 × 10^−5^, FC = −1.27, FDR = 0.0047), *VPS24* (*P* value = 6.20 × 10^−5^, FC = 1.19, FDR = 0.073), *SASH1* (*P* value = 6.90 × 10^−5^, FC = −1.87, FDR = 0.073), *RC3H2* (*P* value = 8.20 × 10^−5^, FC = −1.40, FDR = 0.073), and *TCF4* (*P* value = 1.18 × 10^−4^, FC = −1.78, FDR = 0.075), respectively (i.e., top five genes shown in Table S4 in Supplementary Material). Among them, none was significantly changed in week 12 placebo group at a nominal *P* value < 0.01. Four genes (blue color) were significantly changed in TPM responders compared with TPM non-responders at a nominal *P* value < 5 × 10^−3^ with a |FC| > 1.40 at both weeks 8 and 12, which include *PML* (*P* values = 3.15 × 10^−4^ and 1.10 × 10^−3^, FCs = −1.44 and −1.51, FDRs = 0.24 and 0.12 at weeks 8 and 12, respectively), *SASH1* (*P* values = 1.67 × 10^−3^ and 6.90 × 10^−5^, FCs = −1.49 and −1.87, FDRs = 0.24 and 0.073 at weeks 8 and 12, respectively), *FPR1* (*P* values = 1.11 × 10^−3^ and 5.57 × 10^−4^, FCs = 1.66 and 1.55, FDRs = 0.078 and 0.39 at weeks 8 and 12, respectively), and *GABARAPL1* (*P* values = 3.43 × 10^−3^ and 2.04 × 10^−3^, FCs = 1.44 and 1.48, FDRs = 0.096 and 0.13 at weeks 8 and 12, respectively), and none was significantly changed in placebo responders compared with placebo non-responders at weeks 8 and 12 by using same nominal *P* value and |FC| thresholds.

### Identification of Enriched Pathways in Treatment Responders

**Figure 2:**
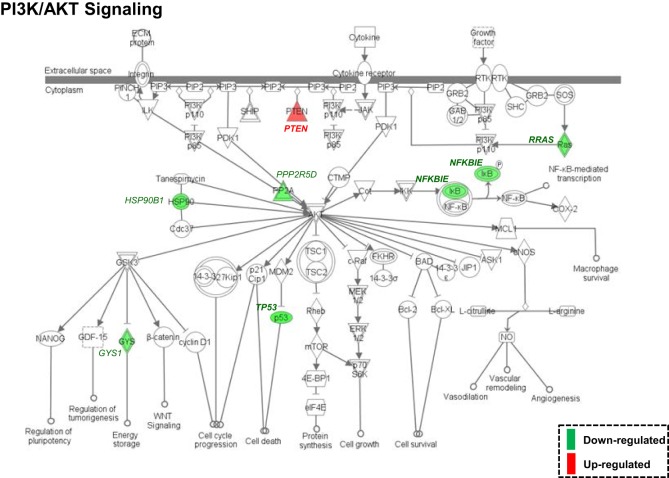
Enriched PI3K/AKT signaling pathway, identified by Ingenuity Pathway Analysis based on 300 differentially expressed genes (nominal *P* values < 0.05) detected exclusively in both week 8 and week 12 topiramate (TPM) groups. Symbols with a single border indicate single genes. Those with a double border indicate complexes of genes or the possibility that alternative genes might act in the pathway. Red color symbols indicate upregulated gene clusters, and green color symbols represent downregulated gene clusters. At both time points, *GYS1, HSP90B1, NFKBIE, PPP2R5D, RRAS*, and *TP53* were consistently downregulated, and *PTEN* was consistently upregulated. If a gene was detected by both the ordinary Student’s *t*-test and the empirical Bayes moderated *t*-test in either the week 8 TPM group or week 12 TPM group, that gene’s corresponding symbol was highlighted in bold font.

**Figure 3:**
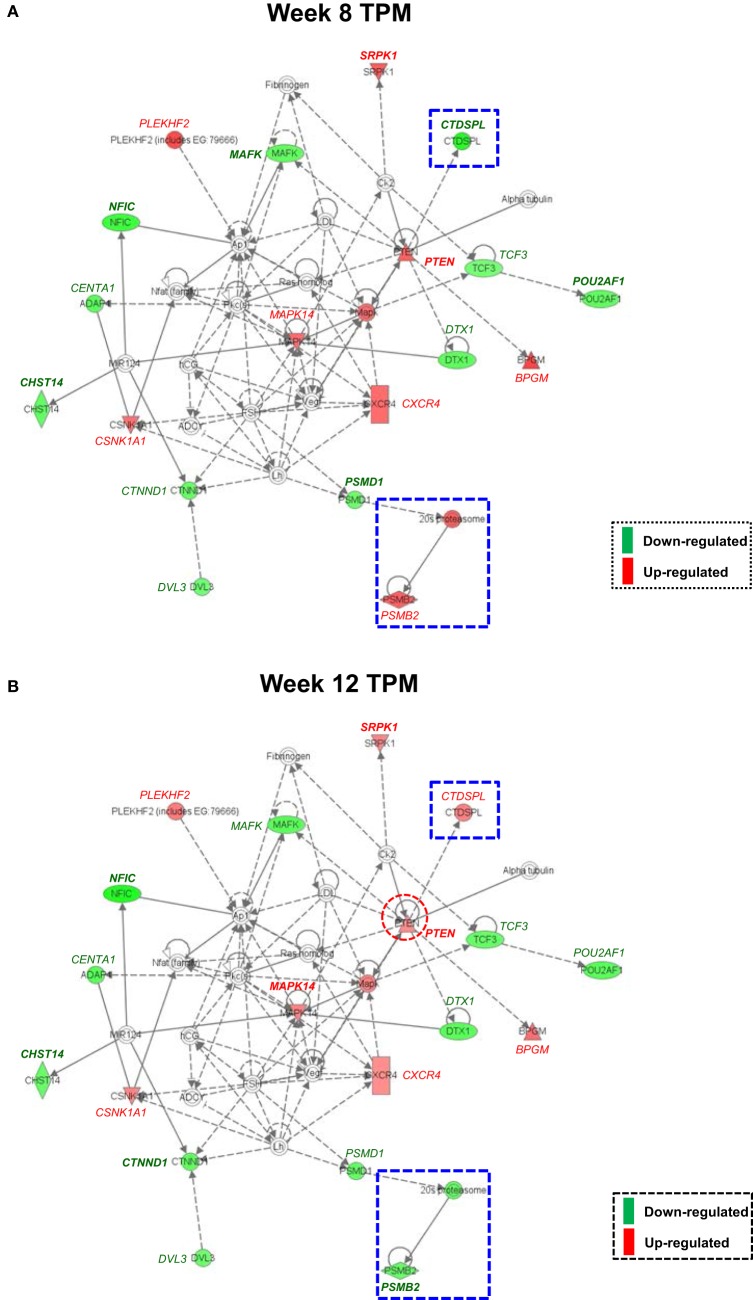
Molecular interaction network revealed by Ingenuity Pathway Analysis detected exclusively for both week 8 and week 12 topiramate (TPM) groups. Solid lines represent direct interactions, and dashed lines represent indirect interactions, with or without filled arrows indicating functional interaction or physical association, respectively. A line with a terminal bar indicates inhibition, whereas filled arrows that are preceded by a terminal bar indicate inhibition as well as functional interaction. Each node’s shape indicates the class of molecule: horizontal ovals are transcription regulators, vertical rectangles are G-protein-coupled receptors, triangles are phosphatases, inverted triangles are kinases, horizontal diamonds are peptidases, double circles are complexes/groups, and single circles are other types of biological molecules. Lines starting and finishing on the same node indicate self-regulation. Arrowheads indicate the directionality of relationship. Nodes are colored according to extent of differential expression, with up- and downregulation represented by red and green colors, respectively. For each group, if a gene was detected by both ordinary Student’s *t*-test and empirical Bayes moderated *t*-test at a nominal *P* value < 0.05, that gene’s corresponding symbol was highlighted in bold font. **(A)** Week 8 TPM (*CENTA1, CHST14, CTDSPL, CTNND1, DTX1, DVL3, MAFK, NFIC, POU2AF1, PSMD1*, and *TCF3* were downregulated and *BPGM, CSNK1A1, CXCR4, MAPK14, PLEKHF2, PSMB2, PTEN*, and *SRPK1* were upregulated, respectively); and **(B)** week 12 TPM (*CENTA1, CHST14, CTNND1, DTX1, DVL3, MAFK, NFIC, POU2AF1, PSMB2, PSMD1*, and *TCF3* were downregulated and *BPGM, CSNK1A1, CTDSPL, CXCR4, MAPK14, PLEKHF2, PTEN*, and *SRPK1* were upregulated, respectively).

Both IPA and Onto-Tools Pathway-Express were applied to detect enriched pathways for 300 and 72 genes uniquely for TPM and placebo responders, respectively. Together, at nominal *P* values < 0.05 and further restricting by FDRs < 0.15 at both time points, 44 enriched pathways were detected for TPM responders, and based on the following selection criteria: (i) number of genes ≥3, and (ii) nominal *P* values < 0.05 at both weeks 8 and 12, and further restricting by FDRs < 0.15 at both time points, 7 selected pathways can be classified into four categories: Neuronal Function/Synaptic Plasticity (protein ubiquitination pathway), Signal Transduction (phosphatidylinositol signaling system and PI3K/AKT signaling), Inflammation/Immune Function (antigen presentation pathway, fMLP signaling in neutrophils, and role of PKR in interferon induction and antiviral response), and Oxidative Stress Response (NRF2-mediated oxidative stress response) (Table 4). Also, two pathways in “Signal Transduction” Category, i.e., Phosphatidylinositol Signaling System and PI3K/AKT Signaling pathways, three pathways in “Inflammation/Immune Function” Category, i.e., Antigen Presentation Pathway, fMLP Signaling in Neutrophils, and Role of PKR in Interferon Induction and Antiviral Response pathways, and the “NRF2-mediated Oxidative Stress Response” pathway in “Oxidative Stress Response” Category, contained >50% genes that were detected by both the ordinary Student’s *t*-test and the empirical Bayes moderated *t*-test in either the week 8 TPM group or week 12 TPM group, highlighting that these pathways have more statistical support for their significance. By applying same selection criteria, no enriched pathways were detected for placebo responders, indicating that these pathways are specific to TPM response. Genes of PI3K/AKT signaling pathway changed exclusively in both week 8 and week 12 TPM responders were shown in Figure 2. Whereas 6 genes, i.e., *GYS1, HSP90B1, NFKBIE, PPP2R5D, RRAS*, and *TP53*, were downregulated, one gene, i.e., *PTEN*, was upregulated, which was also the gene shared between the three genes detected for phosphatidylinositol signaling system and the seven genes detected for PI3K/AKT signaling in the Signal Transduction category (Table 4). Because PTEN, a central negative regulator of the PI3K pathway (41), is required for modulating synaptic activity during plasticity (42), a 36-node *PTEN*-centered molecular interaction network was generated for each of week 8 and week 12 TPM groups (Figures 3A,B), respectively. At week 8, 11 genes, i.e., *CENTA1, CHST14, CTDSPL, CTNND1, DTX1, DVL3, MAFK, NFIC, POU2AF1, PSMD1*, and *TCF3*, were downregulated and 8 genes, i.e., *BPGM, CSNK1A1, CXCR4, MAPK14, PLEKHF2, PSMB2, PTEN*, and *SRPK1*, were upregulated. At week 12, 11 genes, i.e., *CENTA1, CHST14, CTNND1, DTX1, DVL3, MAFK, NFIC, POU2AF1, PSMB2, PSMD1*, and *TCF3*, were downregulated, and 8 genes, i.e., *BPGM, CSNK1A1, CTDSPL, CXCR4, MAPK14, PLEKHF2, PTEN*, and *SRPK1*, were upregulated, respectively. Of the 19 DE genes for week 8 TPM and week 12 TPM groups, it is noted that the majority (89.47%; 17/19) of them have consistent directions of gene expression changes between weeks 8 and 12, and only two genes (i.e., *CTDSPL* and *PSMB2*) had opposite direction of gene expression changes, and such time-dependent change which may be attributed METH-induced inflammation, prolonged TPM exposure by study participants, and other factors. It is also worth mentioning that comparting these two networks at two different time points, the six hub genes [defined as gene nodes each with a degree ≥4 (excluding self-loops)] i.e., *CSNK1A1, CTNND1, CXCR4, MAFK, MAPK14*, and *PTEN*, had consistent directions of gene expression changes for week 8 and week 12 TPM groups, and only two peripheral genes, i.e., *CTDSPL* and *PSMB* (shown in dashed blue rectangles), displayed different directions of changes, indicating that this gene network is relatively stable over time. Among these DE genes, *CSNK1A1, CTNND1, CXCR4, DTX1, MAPK14, PLEKHF2, PSMB2, PSMD1*, and *PTEN* have biologically important roles for TPM responses.

**Table 4:** Significantly enriched pathways for 300 genes consistently detected exclusively in weeks 8 and 12 topiramate (TPM) groups based on secondary outcomes (*n* = 7).^a^

Category/pathway name	Input genes in pathway (#)	*P* value	FDR^e^
**Neuronal function/synaptic plasticity (***n*** = 1)**			
Protein ubiquitination pathway^b^	*ANAPC4, DNAJC17, HSP90B1*, ***PSMB2***, *STUB1, USP38*, ***DNAJC14***, ***PSMD1***, *DNAJC15, USP6*, ***AMFR*** (11)	4.68 × 10^−3^	0.145

**Signal transduction (***n*** = 2)**			
*Phosphatidylinositol signaling system^c^	***INPP5B***, ***PIK3C2B***, ***PTEN*** (3)	9.88 × 10^−8^	3.66 × 10^−6^
PI3K/AKT signaling^b^	***TP53***, *HSP90B1, GYS1*, ***RRAS***, *PPP2R5D*, ***NFKBIE***, ***PTEN*** (7)	4.37 × 10^−3^	0.145

**Inflammation/immune function (***n*** = 3)**			
*Antigen presentation pathway^c^	*CTSB, CANX*, ***TAPBP***, ***HLA-DPA1*** (4)	1.14 × 10^−17^	8.41 × 10^−16^
fMLP signaling in neutrophils^b^	***GNAI2***, ***PIK3C2B***, ***RRAS***, ***NFKBIE***, *ARPC3*, ***GNG2***, ***FPR1*** (7)	2.40 × 10^−3^	0.145
**Role of PKR in interferon induction and antiviral response^b^^,^^d^	***TP53***, ***MAPK14***, ***NFKBIE***, ***TNF*** (4)	4.37 × 10^−3^	0.145

**Oxidative stress response (***n*** = 1)**			
*NRF2-mediated oxidative stress response^b^	***GSTM1***, ***PIK3C2B***, *DNAJC17*, ***GSTM2***, ***MAPK14***, ***SCARB1***, ***RRAS***, ***NQO2***, ***DNAJC14***, *DNAJC15*, ***MAFK*** (11)	2.34 × 10^−4^	0.0640

*^a^Pathways were selected from a total of 44 enriched pathways based on a total of 300 genes detected exclusively for both week 8 and week 12 TPM groups based on the following criteria: detected by either Ingenuity Pathway Analysis (IPA; http://www.ingenuity.com/) or Onto-Tools Pathway-Express (https://bioportal.bioontology.org/projects/Onto-Express) with (i) number of genes ≥3, (ii) nominal *P* values < 0.05 at both weeks 8 and 12, and further restricting by false discovery rates (FDRs) < 0.15 at both time points. Pathways indicated by a single asterisk were statistically significant after Bonferroni correction (i.e., nominal *P* values < 0.05/44 = 1.14 × 10^−3^). A pathway indicated by double asterisks was also detected by primary analysis presented in Ref. (30). A gene that was detected by both the ordinary Student’s *t*-test and the empirical Bayes moderated *t*-test in either the week 8 TPM group or week 12 TPM group was highlighted in bold font. The respective *t*-test’s *P* values in week 8 TPM and week 12 TPM groups for genes contained in each of the seven enriched pathways are shown in Table S6 in Supplementary Material*.

*^b^Detected by IPA from Ingenuity Pathways Knowledge Base*.

*^c^Detected by Onto-Tools Pathway-Express from Kyoto Encyclopedia of Genes and Genomes*.

*^d^Pathways shared with those detected for the primary efficacy outcome*.

*^e^FDR was estimated by the Benjamini–Hochberg method*.

## Discussion

The advent of global gene expression profiling has generated unprecedented insight into our molecular understanding of drug addiction and treatment. We previously have identified genes involved in glutamate receptor and GABA receptor signaling are changed among TPM responders compared with non-responders based on primary outcome (i.e., METH abstinence) and a set of crucial pathways involved in neuronal function/synaptic plasticity, signal transduction, cardiovascular function, and inflammation/immune function are significantly enriched among TPM responders (30). However, the primary analysis was limited to only METH abstinence phenotype (30), and certain significantly modulated genes and pathways could be missed because of the limited sample sizes. This study differed from the primary analysis in several aspects. First, the secondary analysis applied the LCA classifications based on the six non-longitudinal binary secondary outcomes of weeks 1–12 into “responder” and “non-responder” classes, and those in the “responder” class scored consistently better on each of the six secondary outcomes than those in the “non-responder” class, as shown in Ma et al. (31). Such a classification provided greater sample sizes and more balanced comparisons for week 8 TPM, week 8 placebo, week 12 TPM, and week 12 placebo groups, compared with the primary analysis. Second, this secondary analysis applied both ordinary Student’s *t*-test and the empirical Bayes moderated *t*-test, which is distinct from the more traditional association analyses utilizing only the ordinary Student’s *t*-test, and is particularly suitable for small-to-moderate samples sizes. Although the results obtained from this study are from peripheral blood and need further validation and examination in CNS, it is quite striking that our secondary analyses based on six non-longitudinal binary secondary outcomes revealed a rather consistent and meaningful pattern that TPM changed more genes than placebo at both week 8 and week 12, which not only confirmed important genes and pathways revealed by primary analysis based on primary outcome (30) but also extended the results by identifying additional new genes and pathways on TPM response to METH addiction.

At individual gene level, because gene sets detected by both ordinary Student’s *t*-test and empirical Bayes moderated *t*-test had relatively high proportions of overlap (64.50 ± 7.43%, range 56.55–74.31%), they were merged together at each time point for TPM and placebo groups, respectively (Table 1). Among 300 genes detected exclusively in TPM responders at weeks 8 and 12, 34 genes had nominal *P* values < 5 × 10^−3^ at either time point and FDRs < 0.15 at both time points. After excluding two genes with inconsistent directions of gene expression changes, i.e., *FNIP2* and *TOX4*, 32 genes were selected based on the above statistics criteria with consistent directions of gene expression changes at both week 8 and week 12 (Table 2). FDR is expected proportion of erroneously rejected null hypotheses among rejected ones. FDR threshold is determined from observed *P* value distribution, and hence is adaptive to actual data. An FDR threshold of 0.15 was chosen as significance threshold, which has been used in previous gene expression studies in choosing significant genes, e.g., Ref. (43–48). FDR is defined as the expected number of discoveries that are not truly DE divided by the total number of discoveries. An overly stringent control for FDR can result in a large number of false negatives (49–51). Therefore, determination of an appropriate FDR threshold is critical for effectively identifying truly DE genes, while minimizing both false positives and false negatives. By applying a cross-validation approach, an optimal selection of FDR threshold is shown to provide a good performance on model selection and prediction (52). Twelve of them, i.e., *CASP4, COX19, CUX1, GABARAPL1, GNG2, GPR155, HSF1, IL15RA, NLRP1, SIL1, SLC25A19*, and *UBAP2*, could have critical functions for drug addiction. Of them, *GABARAPL1* encodes a protein (87% identical, 94% similar) very similar to GABA_A_-receptor-associated protein (GABARAP) (53) and is expressed at higher levels than *GABARAP* in CNS (54). Further, GABARAPL1 is suggested to be a major protein interacting with GABA_A_ receptors (54). *GPR155*, which encodes an integral membrane protein related to G-protein-coupled receptors (GPCRs), is highly expressed in lateral part of striatum and hippocampus (55). Many neurons that are identifiable as GABAergic might express *GPR155*, implicating its pivotal role in GABAergic neurotransmission (55). *GPR155* is dysregulated in lymphoblastoid cells in males with autism spectrum disorders (ASDs) relative to their non-affected siblings, suggesting that the gene is associated with ASD (56). IL15, an important cytokine in immune function, is essential to maintain neurochemical homeostasis (57). *IL15RA*, which encodes IL15 receptor alpha subunit, has a regulatory function during inflammation. *Il15ra* knockout mice have deficits in hippocampal-dependent memory and GABA transmission (58). Thus, *GABARAPL1, GPR155*, and *IL15RA* in GABA receptor signaling could be direct targets for TPM. By contrast, no genes were identified in placebo responders using same criteria.

In this study, we applied both an ordinary Student’s *t*-test and an empirical Bayes moderated *t*-test. The ordinary Student’s *t*-test is the most commonly used method for comparing the expression levels of genes between two groups, and the computation of the *P* value for this test is straightforward as long as the assumptions of the test are satisfied. This test has been used in our previous gene expression study based on the primary outcome (30). The empirical Bayes moderated *t*-test, available in LIMMA package of Bioconductor, is also a popular method for two-group comparisons of gene expressions, which reduces estimated sample variances toward a pooled estimate, producing more stable result when the sample sizes are small (59). Each of ordinary Student’s *t*-test and empirical Bayes moderated *t*-test has distinct advantages and disadvantages. The ordinary Student’s *t*-test is easy to apply, but variance estimates could be skewed by those genes having a very low variance (60). The empirical Bayes moderated *t*-test is an innovative method that borrows information between genes using an empirical Bayes method to obtain posterior variance estimators, and compute a moderated *t* statistic that follows a *t* distribution with augmented degrees of freedom (61), but in this study, this method produces overall higher FDRs compared with ordinary Student’s *t*-test, indicating a lower statistical power. Nevertheless, the two analyses by ordinary Student’s *t*-test and empirical Bayes moderated *t*-test demonstrated largely consistently detected genes. Similar to our study, Uusküla et al. (35) applied two different statistical approaches, i.e., ANOVA and empirical Bayes moderated *t*-test, and genes identified by these two approaches were shown to be relevant for the clinical outcome. We did not choose to apply nonparametric Wilcoxon rank-sum (i.e., Mann–Whitney *U* test) because such test has a reduced statistical power and typically detects fewer genes compared with parametric test (62).

By comparing these above DE genes detected at nominal *P* values < 0.01 based on secondary outcomes with those DE detected at same statistical significance threshold based on primary outcome (30), 55, 11, 11, and 15 genes were shared between primary outcome and secondary outcomes for the week 8 TPM, week 8 placebo, week 12 TPM, and week 12 placebo groups, respectively (Table 3). Among the 55 genes shared between primary outcome and secondary outcomes for week 8 TPM, 9 genes, i.e., 4 upregulated genes, i.e., *CD164, AKAP11, FGFR1OP2*, and *PTEN*, and 5 downregulated genes, i.e., *EMILIN2, DGCR14, BCR, GANAB*, and *NAGK*, were also among the 93 (48 up- and 45 downregulated) representative genes selected based on primary outcome with consistent directions (30). Therefore, these nine DE genes could be particularly related to TPM treatment response for METH dependence. Of them, *PTEN*, which encodes a protein that functions as a protein tyrosine phosphatase as well as a lipid phosphatase, is of particular interest. PTEN plays a critical role in both CNS development and maintenance of CNS circuit structure and function (63). PTEN is shown to be a mediator of synaptic plasticity in the adult brain (42, 64) that is required for NMDAR-dependent long-term depression, and alternations of PTEN at synapses could lead to behavioral and cognitive dysfunctions (65).

At week 8, *VISA* (FDR = 0.0039), *CHST14* (FDR = 0.026), *GAS2L1* (FDR = 0.026), and *ITGA2B* (FDR = 0.026) were exclusively changed by TPM, and at week 12, *ASXL1* (FDR = 0.0047), *VPS24* (FDR = 0.073), *SASH1* (FDR = 0.073), and *TCF4* (FDR = 0.075) were exclusively changed by TPM, respectively. Based on nominal *P* values < 5 × 10^−3^ and |FC| > 1.40, *PML, SASH1, FPR1*, and *GABARAPL1* were exclusively changed by TPM at both at week 8 and week 12. Of them, *FPR1*, which encodes the *N*-formyl peptide receptor, is a GPCR (66) belonging to the top 50 DE genes in the CNS of schizophrenic patients with long durations of illness (67). *GAS2L1* is a susceptibility locus for schizophrenia (68), and *SASH1* gene expression was affected by a history of substance dependence/abuse (69). *TCF4*, which encodes a basic helix-turn-helix transcription factor, regulates gene expression in immune system and in brain development (70) and is significantly associated with schizophrenia (71).

At pathway level, seven biologically important pathways (i.e., Protein Ubiquitination Pathway, Phosphatidylinositol Signaling System, PI3K/AKT Signaling, Antigen Presentation Pathway, fMLP Signaling in Neutrophils, Role of PKR in Interferon Induction and Antiviral Response, and NRF2-mediated Oxidative Stress Response) were significantly enriched in TPM responders compared with non-responders for secondary outcomes (Figure 2). Of them, PI3K/AKT signaling pathway appears to be an important pathway for neuronal survival (72). Six genes (i.e., *GYS1, HSP90B1, NFKBIE, PPP2R5D, RRAS*, and *TP53*) were downregulated by TPM in this pathway, and *HSP90B1*, which encodes an endoplasmic reticulum chaperone gene, is altered in the postmortem brain of bipolar disorder patients (73), and is associated with bipolar disorder (74) and schizophrenia (75–78). *PTEN*, which encodes a dual-specificity protein phosphatase that negatively regulates the PI3K/AKT signaling pathway (79–81), was upregulated by TPM. *PTEN*-centered networks of 36 interactors for week 8 and week 12 TPM responders included 19 dysregulated genes (Figures 3A,B). Of them, *CSNK1A1, CTNND1, CXCR4, DTX1, MAPK14, PLEKHF2, PSMB2, PSMD1*, and *PTEN* could be important for TPM responses. Besides *PTEN, MAPK14*, upregulated at both time points, encodes p38 MAPK, which plays an essential role in ROS formation and oxidative stress (82), production of inflammation mediators (83, 84), and neuronal apoptosis (85, 86). Further, *CXCR4*, also upregulated at both time points, encodes a chemokine receptor critical for anti-inflammatory response (87) and p53-mediated neuronal survival (88). Ubiquitin-mediated proteolysis is involved in the turnover of many short-lived regulatory proteins. Timed destruction of cellular regulators by the ubiquitin–proteasome pathway plays a critical role in ensuring normal cellular processes. Genetic approaches or pharmacological intervention that alters the half-lives of these cellular proteins may have wide therapeutic potential (89). It is worthy of noting that PI3K/AKT signaling and PTEN signaling pathways were detected to be enriched pathways for gene modulated by nicotine (90), and therefore, PI3K/AKT signaling appears to be a crucial pathway affected by various psychoactive drugs.

**Figure 4:**
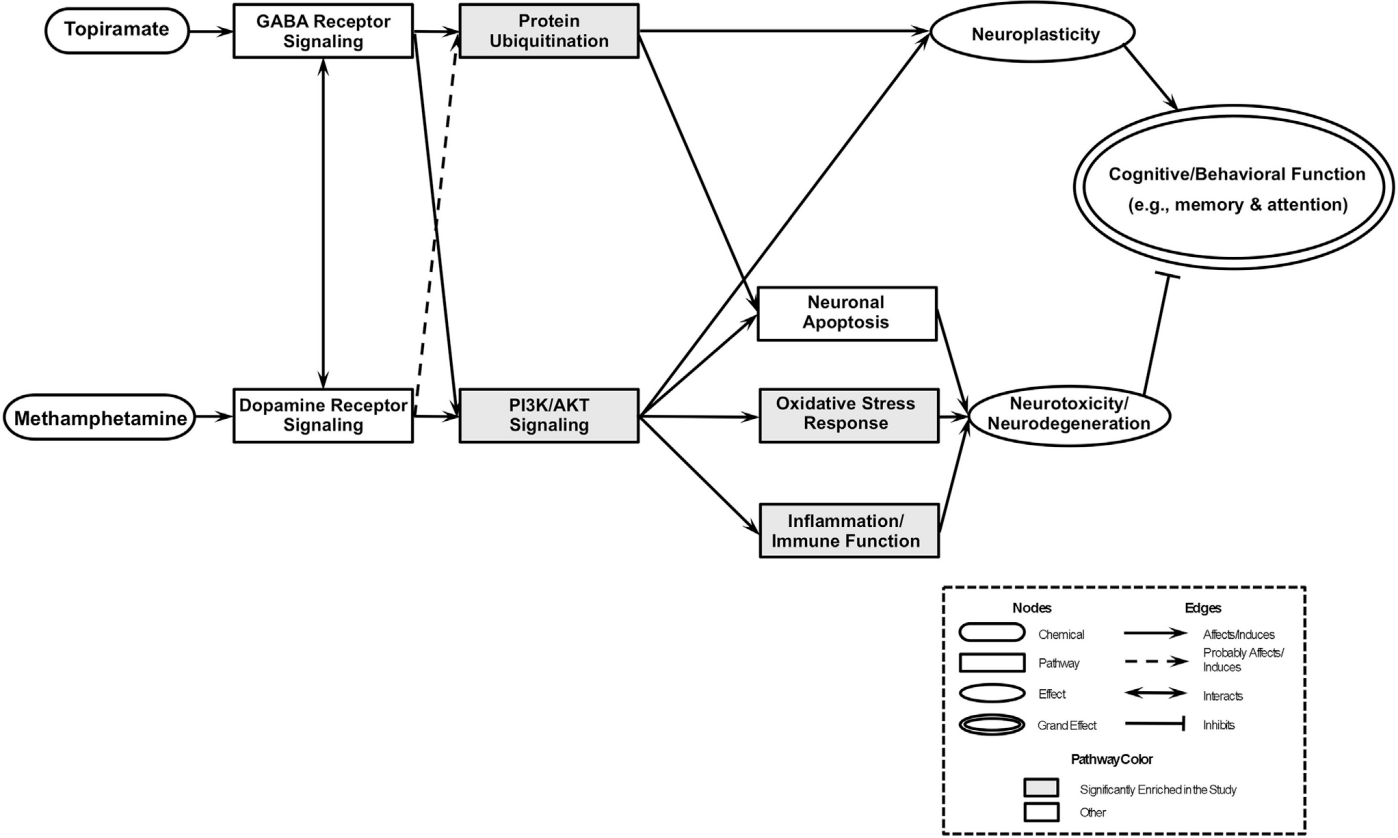
An integrated model of biological pathways related to topiramate (TPM) treatment for methamphetamine (METH) addiction. The joint effects of TPM and METH act on several molecular pathways that eventually lead to modulations of neuroplasticity and neurotoxicity/neurodegeneration, which results in abstinence and reduction of METH use. Pathways enriched exclusively in TPM responder groups at weeks 8 and 12 are highlighted in gray.

An integrative model based on enriched pathways has been proposed to explain the molecular mechanisms of TPM’s effect on METH addiction (Figure 4). METH could lead to increased oxidative stress by altering PI3K/AKT signaling pathway, by interacting with vesicular monoamine transporter 2, which leads to accumulated cytoplasmic dopamine with resultant free radical formation (91). The transcription factor NRF2, a guardian of redox homeostasis, regulates a coordinated induction of a set of cytoprotective, antioxidant, and anti-inflammatory genes in response to oxidative stress and inflammation (92, 93). NRF2-mediated oxidative stress response pathway is one of the major intrinsic antioxidant response of the brain, and NRF2 is a therapeutic target for treatment of neurodegenerative diseases, e.g., Alzheimer’s disease and Parkinson’s disease (94). TPM provides neuroplasticity by modulating protein ubiquitination and PI3K/AKT signaling pathways, which in turn, could decrease oxidative stress and increase neuroplasticity, which then could lead to abstinence and reduction of METH use. The integrative model proposed based on primary outcome [i.e., Figure 2 of Ref. (30)] included “PI3K/Akt/GSK-3 signaling” and “Mitochondrial oxidative stress” pathways downstream of “Dopamine receptor signaling” pathway, and the integrative model for this study (i.e., based on secondary outcomes) has substantiated the previous models branch downstream of “Dopamine receptor signaling,” and further the “Protein Ubiquitination” pathway is a newly added pathway downstream of “GABA Receptor Signaling” pathway of the previous integrative model, which then could affect both neuroplasticity and neuronal apoptosis.

In this study, by analyzing gene expression profiling of whole blood, we attempted to define transcriptional patterns that differentiate TPM responders from non-responders. Whole blood has been increasingly used as a more accessible tissue for identifying proxy gene expression biomarkers for CNS, e.g., brain’s circadian phase (95) and ASD (96, 97). As shown in Vawter et al. (98), the use of whole blood for studying gene expression could avoid several important confounding variables associated with postmortem brain studies, e.g., hypoxia, pyrexia, postmortem interval, mRNA integrity, cellular heterogeneity of subcortical and cortical tissues that need to be controlled for in subsequent gene expression analyses. Further, recent studies demonstrate that blood cell-derived RNA could be used to distinguish schizophrenia, bipolar disorder, and control samples with high accuracies (99). The consistency of peripheral gene expression data and the overlap with brain expression has also been evaluated by Rollins et al. (100), which demonstrated that postmortem subjects’ brain and peripheral blood mononuclear cell (PBMC) profiles showed co-expression levels of summarized transcripts for 4,103 of 17,859 (22.9%) RefSeq transcripts. However, because of the concern that whole blood gene expression is not completely correlated with brain gene expression and has a heterogeneous composition (e.g., T-and B-lymphocytes, PBMCs, and other cell types), gene expression patterns identified by this study shall be interpreted with caution and required to be further validated in CNS studies. One of the major limitations of this study is that no quantitative real-time PCR was applied to validate gene expression changes of the 32 gene candidates presented in Table 2, which was primarily due to a lack of sufficient high quality RNA for us to conduct such analysis.

In conclusion, this study of the transcriptome of secondary outcomes provided additional biological insights into TPM treatment response for METH dependence beyond the previous gene expression study based the primary outcome (30). Analyses based on ordinary Student’s *t*-test and empirical Bayes *t*-test have not only identified novel sets of genes consistently for week 8 and week 12 TPM responders, but also detected several unique pathways, particularly protein ubiquitination and PI3K/AKT signaling pathways, and also a novel PTEN-centered gene interaction network. Therefore, TPM treatment could lead to a decreased METH dependence by reducing oxidative stress and inflammation and enhancing neuroplasticity, which have extended the integrative model based on primary outcome. Combining results obtained from this study with those of the previous study (30), TPM response in METH-dependent subjects is a highly complex process encompassing a diverse spectrum of biological pathways that can be classified into Neuronal function/Synaptic plasticity, Signal transduction, Cardiovascular function, Inflammation/Immune function, and Oxidative Stress Response categories.

## Ethics Statement

The Institutional Review Boards of each clinical site and the VA Human Rights Committee approved the protocol for and conduct of the study.

## Author Contributions

Conceived, designed, and performed the experiments: TN, JW, JM, and MDL. Analyzed the data: TN, JL, JW, and JM. Contributed reagents/materials/analysis tools: TN, JW, and MDL. Wrote the paper: TN, JW, and MDL.

## Conflict of Interest Statement

The authors declare that the research was conducted in the absence of any commercial or financial relationships that could be construed as a potential conflict of interest.

## Footnotes

^1^https://www.qiagenbioinformatics.com/products/ingenuity-pathway-analysis/.

^2^https://bioportal.bioontology.org/projects/Onto-Express. Note: The original Onto-Express has been developed to iPathwayGuide (Advaita, Plymouth, MI) at website: https://www.advaitabio.com/gene-ontology-pathway-analysis.html.

^3^http://www.ncbi.nlm.nih.gov/geo/query/acc.cgi?acc=GSE107015.
