# Supplementary material for: Identification of Novel Signal Transduction, Immune Function, and Oxidative Stress Genes and Pathways by Topiramate for Treatment of Methamphetamine Dependence Based on Secondary Outcomes
Source: Front Psychiatry. 2017 Dec 13;8:271. doi: 10.3389/fpsyt.2017.00271 (PMC5733474; doi:10.3389/fpsyt.2017.00271)
Supplement: Supplementary file 1 [file Presentation_1.PDF]

SUPPLEMENTARY TEXT S1

The 6 non-longitudinal binary secondary outcome measures include SOC112, SOD112, SOE112, SOF112, SOG112, and SOH112, which are defined in the following:

(i) Secondary Outcome C for Weeks 1-12 (SOC112): The proportion of subjects with 21 consecutive days of METH abstinence during weeks 1 through 12 (study days 1 through 84) during which all urine drug screens must be METH-free. I.e., this is a binary outcome such that each study participant has a score of either 0 (< 21 consecutive days of METH abstinence) or 1 (≥ 21 consecutive days of METH abstinence) during weeks 1 through 12 based on urine drug screens only.

(ii) Secondary Outcome D for Weeks 1-12 (SOD112): The proportion of subjects with 21 consecutive days of METH abstinence during weeks 1 through 12 (study days 1 through 84) during which time all urine drugs screens must be METH-free and there is no self-report of METH use. I.e., this is a binary outcome such that each study participant has a score of either 0 (< 21 consecutive days of METH abstinence) or 1 (≥ 21 consecutive days of METH abstinence) during weeks 1 through 12 based on urine drug screens and self-report.

(iii) Secondary Outcome E for Weeks 1-12 (SOE112): The proportion of subjects who decrease the overall proportion of positive METH use days by the Substance Use Report (SUR) during weeks 1 through 12 (study days 1 through 84) by 25% or more of their self-reported METH use in the 14-day baseline period. I.e., this is a binary outcome such that each study participant has a score of either 0 (attaining a decrease of < 25% positive METH use days among the total number of days during weeks 1 through 12 giving non-missing self-reports on METH use in comparison with self-reported METH use in the 14-day baseline period) or 1 (attaining a decrease of ≥ 25% positive METH use days among the total number of days during weeks 1 through 12 giving non-missing self-reports on METH use in comparison with self-reported METH use in the 14-day baseline period).

(iv) Secondary Outcome F for Weeks 1-12 (SOF112): The proportion of subjects who decrease the overall proportion of positive METH use days by the Substance Use Report (SUR) during weeks 1 through 12 (study days 1 through 84) by 50% or more of their self-reported METH use in the 14-day baseline period. I.e., this is a

binary outcome such that each study participant has a score of either 0 (attaining a decrease of < 50% positive METH use days among the total number of days during weeks 1 through 12 giving non-missing self-reports on METH use in comparison with self-reported METH use in the 14-day baseline period) or 1 (attaining a decrease of ≥ 50% positive METH use days among the total number of days during weeks 1 through 12 giving non-missing self-reports on METH use in comparison with self-reported METH use in the 14-day baseline period).

(v) Secondary Outcome G for Weeks 1-12 (SOG112): The proportion of subjects who decrease the median of METH quantitative urine concentration during weeks 1 through 12 (study days 1 through 84) by 25% or more of their median methamphetamine quantitative urine concentration in the 14-day baseline period. I.e., this is a binary outcome such that each study participant has a score of either 0 (attaining a decrease of < 25% for the median METH quantitative urine concentration during weeks 1 through 12 in comparison with the median METH quantitative urine concentration in the 14-day baseline period) or 1 (attaining a decrease of ≥ 25% for the median METH quantitative urine concentration during weeks 1 through 12 in comparison with the median METH quantitative urine concentration in the 14-day baseline period).

(vi) Secondary Outcome H for Weeks 1-12 (SOH112): The proportion of subjects who decrease the median of METH quantitative urine concentration during weeks 1 through 12 (study days 1 through 84) by 50% or more of their median methamphetamine quantitative urine concentration in the 14-day baseline period. I.e., this is a binary outcome such that each study participant has a score of either 0 (attaining a decrease of < 50% for the median METH quantitative urine concentration during weeks 1 through 12 in comparison with the median METH quantitative urine concentration in the 14-day baseline period) or 1 (attaining a decrease of ≥ 50% for the median METH quantitative urine concentration during weeks 1 through 12 in comparison with the median METH quantitative urine concentration in the 14-day baseline period).
